# Low-Grade Chondrosarcoma of the Proximal Phalanx: A Rare Presentation

**DOI:** 10.1155/2019/6402979

**Published:** 2019-03-14

**Authors:** Paul Knapp, Alberto Aviles, Christopher Najarian

**Affiliations:** ^1^St. John Macomb-Oakland Hospital, Madison Heights Campus, 27351 Dequindre Rd, Madison Heights, MI 48071, USA; ^2^St. John Providence Health System, 11900 E Twelve Mile Rd, Suite #308, Warren, MI 48093, USA

## Abstract

**Case:**

Chondrosarcomas are the second most common primary malignant sarcoma of the bone, though it is overall a rare tumor. Our case presents a 36-year-old Caucasian male who complained of an enlarging mass at his third finger MCP joint. After assessing the full clinical scenario, it was determined that wide excision with ray resection would provide the best result for this patient.

**Conclusion:**

This study explains a rare malignancy presenting in the proximal phalanx of the hand. The following report will review chondrosarcomas involving the hands, help differentiate between low-grade chondrosarcomas and enchondromas, and briefly cover treatment modalities.

## 1. Introduction

Chondrosarcomas are malignant tumors made up of cartilage-producing cells. Of all the malignant bone tumors, these make up around 20% [1]. The peak incidence of these tumors arises in the fifth and sixth decade [2]. Sex prevalence has been found to be nearly equal [1]. These tumors are broken down into three main histological grades: low (Grade 1), intermediate (Grade 2), and high (Grade 3) with a majority of these lesions falling into the low-grade category [1]. In a literature review performed by Stomeo et al., they were able to describe five key histologic features of these lesions which included (1) production of malignant cartilage, (2) medullary cavity infiltration, (3) osseous trabeculae entrapment, (4) Haversian system infiltration, and (5) normal bone destruction [3]. Low-grade chondrosarcomas demonstrate a predominance of mild hypercellularity and mild atypia compared to significant hypercellularity and pleomorphism with higher grade tumors [4].

About 70-75% of these tumors arise in the pelvis, femur, and humerus, and albeit very rare, it has been found that chondrosarcomas are actually the most common primary malignant tumor in the digits [4]. In a study by Ogose et al., of all chondrosarcomas in the hands and feet, the fifth finger and calcaneus were the most common sites [5]. One of the more challenging differentiations in the realm of orthopedic oncology is that of enchondroma and low-grade chondrosarcoma because of their similar radiographic and histologic appearance. This determination is critical for the proper treatment modality and for decreasing the rate of recurrence. Based on our literature review, there were about fifty articles found covering chondrosarcomas in the hand and considerably fewer involving the proximal phalanx.

## 2. Informed Consent

The patient was informed that information from the case would be submitted for publication. The patient understood and consented to the use of all data, photographs, and other health information.

## 3. Case Summary

**Figure 1:**
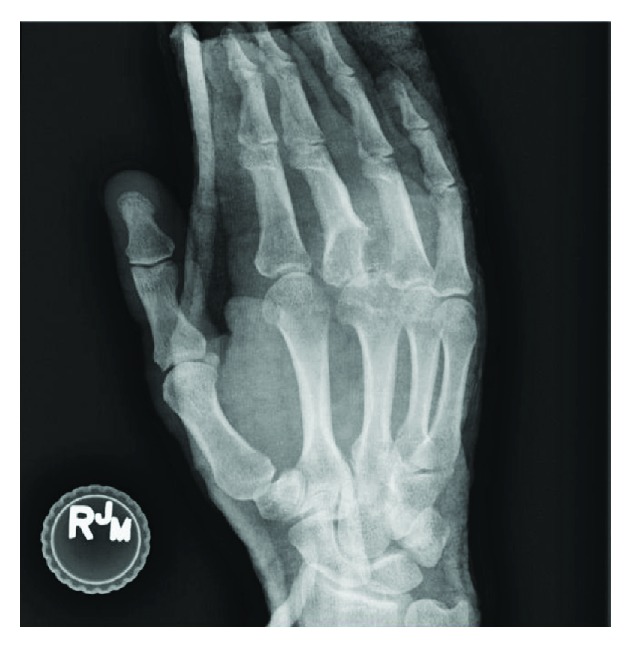
Oblique radiograph of the right hand demonstrating a lytic destructive process of the base of the proximal phalanx, third digit.

**Figure 2:**
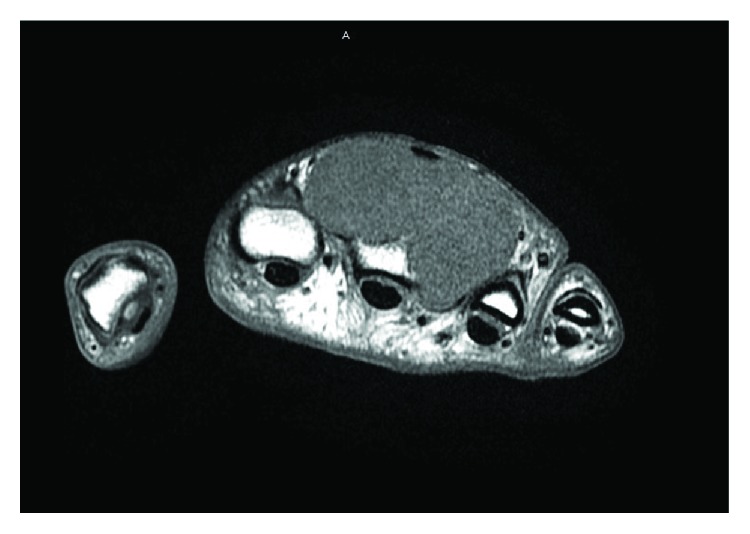
T1 axial MRI image of the right hand demonstrating an expansile destructive mass involving the proximal phalanx of the third digit measuring 3.8 × 2.2 × 3.5 cm.

**Figure 3:**
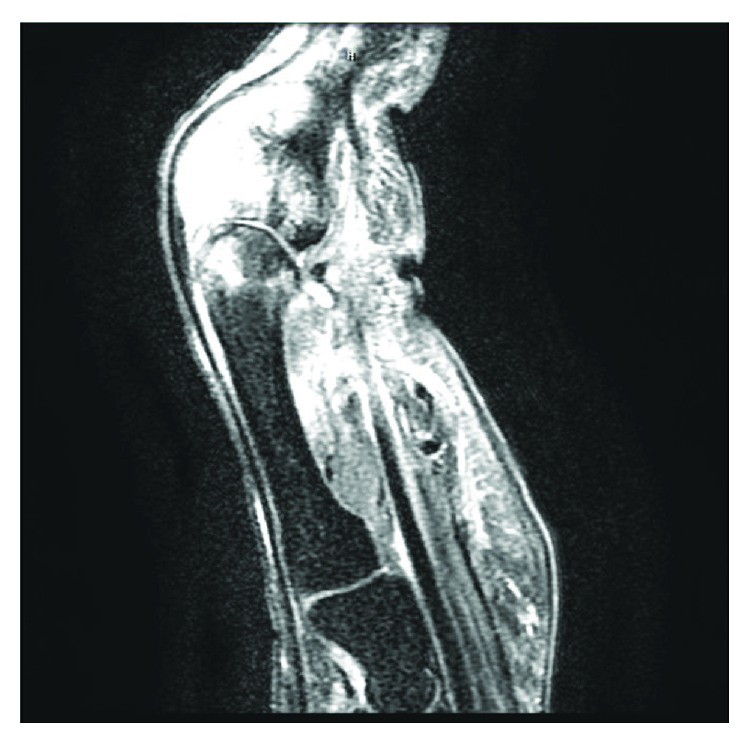
T2 sagittal MRI image of the described chondrosarcoma.

**Figure 4:**
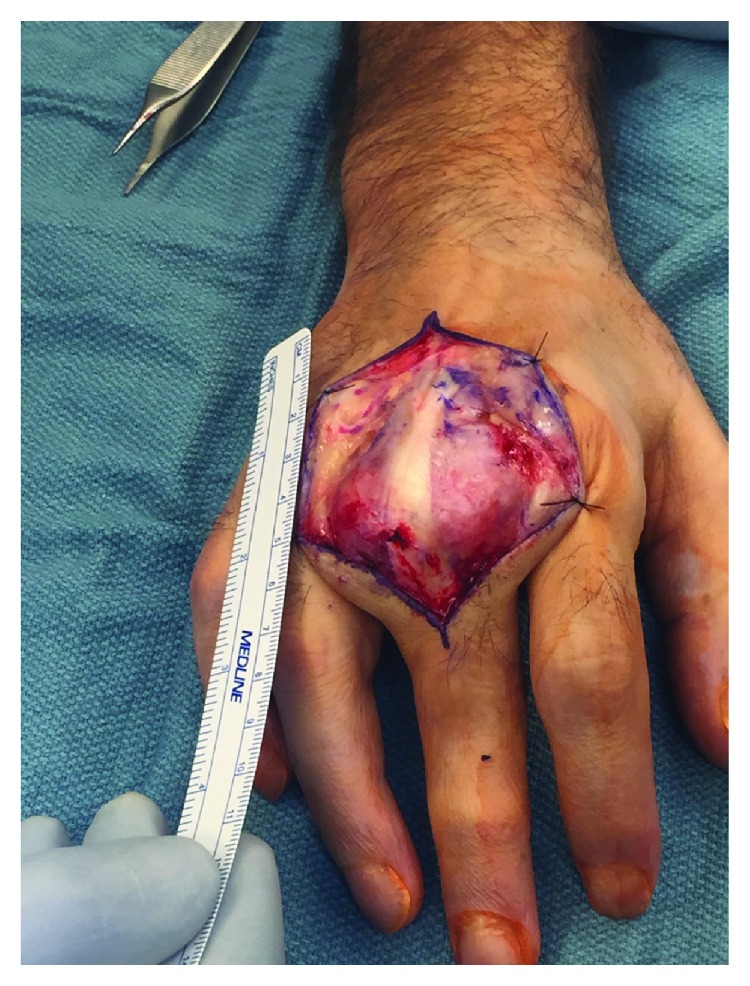
Gross surgical image of dissection/approach.

**Figure 5:**
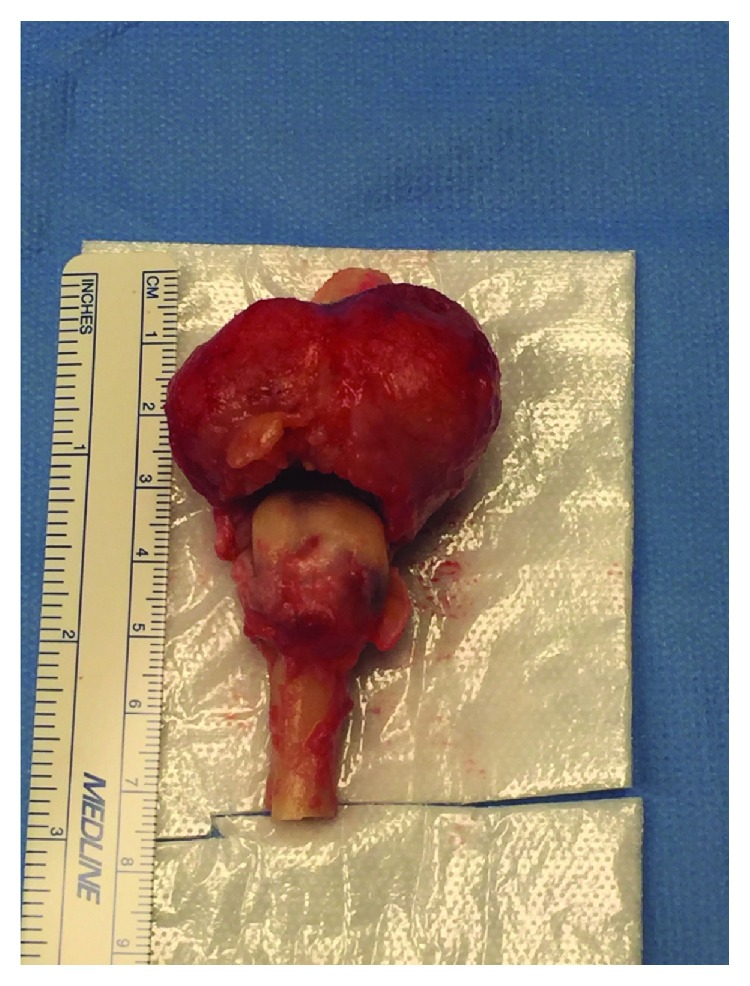
Gross surgical image of excised tumor specimen.

**Figure 6:**
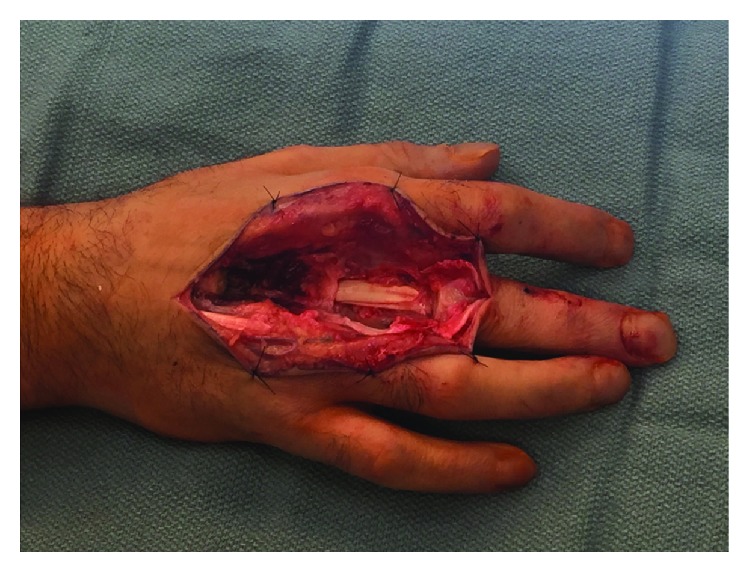
Gross surgical image of dead space after ray resection/tumor excision.

**Figure 7:**
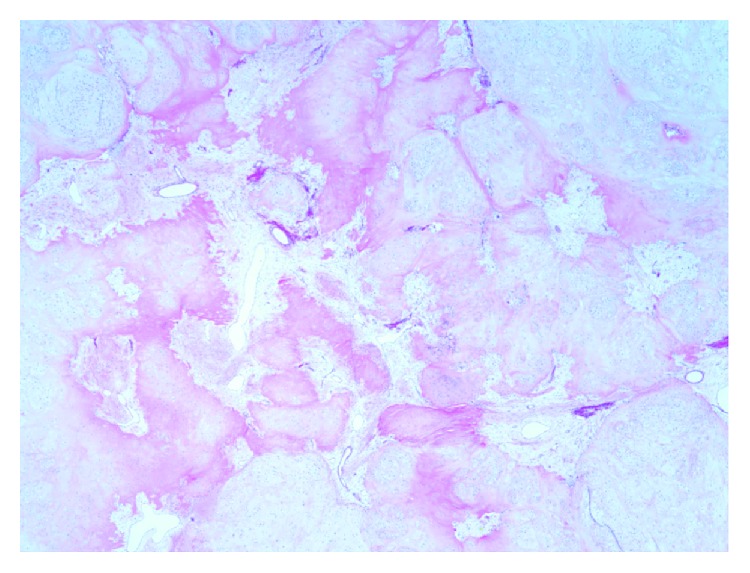
Microscopic image of chondrosarcoma at 10x magnification.

**Figure 8:**
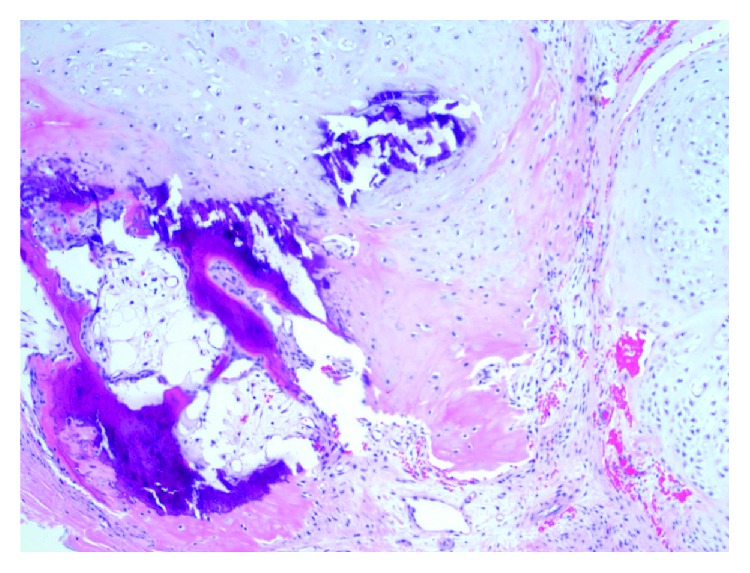
Microscopic image of chondrosarcoma at 20x magnification.

**Figure 9:**
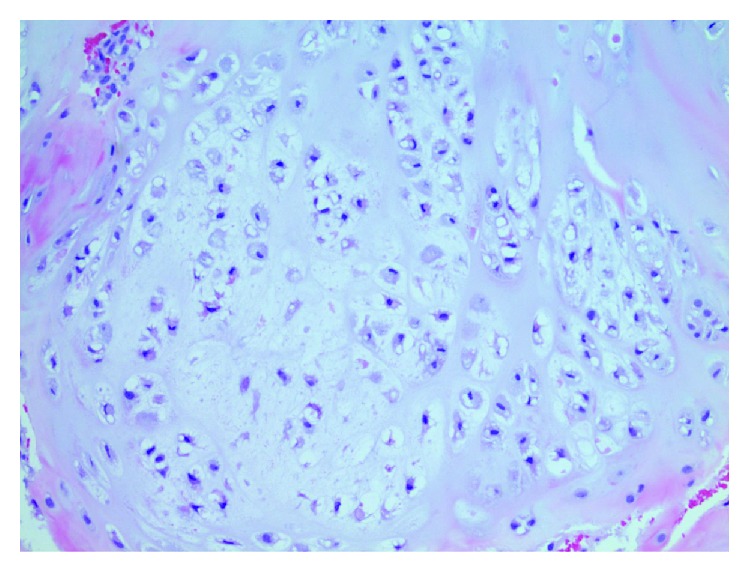
Microscopic image of chondrosarcoma at 40x magnification.

**Figure 10:**
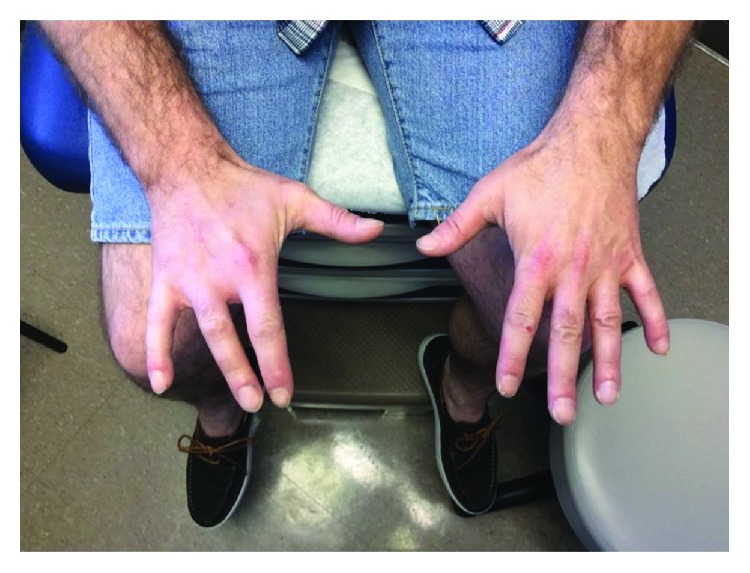
Image of patient's hand at one-year follow-up visit.

Our case describes a 36-year-old Caucasian male with no significant medical history who complained of a painful mass over the dorsum of his third digit metacarpophalangeal (MCP) joint and long finger. According to the patient, it had been present since childhood but was recently becoming a limiting factor in his work and activities of daily living. The patient had been unable to fully flex the affected digit for years but recently began to lose mobility of neighboring digits of the hand. Radiographs obtained demonstrated a lytic lesion within the head of the proximal phalanx of the third digit with surrounding periosteal reaction (Figure 1). MRI was also obtained to determine extent of the tumor and is shown in Figures 2 and 3. Incisional biopsy was performed which confirmed Grade 1 chondrosarcoma. The decision was made for the patient to undergo tumor resection with long finger amputation and metacarpal ray resection. Expectations were explained in detail for the patient prior to surgery, including his future function and cosmetic appearance. The patient underwent the procedure successfully and without any complications. The diseased portions of the proximal phalanx of the long finger and third metacarpal head were resected and sent to pathology to confirm chondrosarcoma and to determine appropriate surgical margins. These were also sent from our institution to a larger neighboring institution's pathology department for verification. The resection was completed down to the level of the midshaft of the third metacarpal, and the remainder of the long finger was removed. The intermetacarpal ligaments and dorsal interossei from the second and fourth digits were approximated to obliterate the dead space remaining (see Figures 4–6). The pathology department at our institution was also contacted, and the pathology slides (10x, 20x, and 40x, respectively) were provided to us for reference and are seen in Figures 7–9. The patient was discharged home with instructions to follow-up in 2 weeks, and in the interim, begin hand therapy for the remaining digits. He was followed closely on an outpatient basis to monitor for any recurrence and assess his function in the continued post-op period. At the first post-op visit, the patient had normal postoperative soreness but severe pain and limitation in motion of neighboring digits was resolved. At one year follow-up, the patient had excellent function/strength in the hand without any signs of recurrence. The patient was not endorsing any pain in the hand and states that he was able to return to work without any limitations (see Figure 10).

## 4. Discussion

This case report aims at summarizing the intricacies of diagnosis and treatment options with low-grade cartilage lesions. Chondrosarcomas can be classified as either primary or secondary based upon a preexisting lesion. In secondary chondrosarcomas, there are preexisting benign cartilage tumors that have transformed into malignant lesions. According to a review article by Lin et al., the authors were able to find that the majority (about 88%) of cases stemmed from osteochondromas, with solitary more common than hereditary multiple exostosis (HME). Additionally, there were documented cases of secondary chondrosarcomas from patients with Ollier disease, Maffucci syndrome, and more rarely from solitary enchondroma, synovial chondromatosis, and chondromyxoid fibroma [6]. Unfortunately in our case, there was no previous workup of this patient's lesion, so we were unable to determine whether it was primary or secondary in nature.

The differentiation between an enchondroma and a low-grade chondrosarcoma has become one of the more challenging scenarios in the field of orthopedic oncology. These cases must involve a multidisciplinary approach between orthopedics, pathology, and oncology because of their similar clinical and histologic presentation. Importance of this distinction is related to determining the proper treatment course. Enchondromas are benign lesions while chondrosarcomas have a risk of metastasis depending solely on the grade of the lesion [4]. In a study done by Ferrer-Santacreu et al., the authors were able to summarize the radiologic, pathologic, and clinical differences between low-grade chondrosarcomas and enchondromas. Notable clinical characteristics that favored a diagnosis of chondrosarcoma included patient age > 25 years, axial skeleton location, inflammatory pain, evidence of periosteal reaction and endosteal scalloping on radiographs, and pathology demonstrating a single mass with probable bone marrow invasion [7].

Typically, cartilage lesions in the small bones of the hands and feet are found to be enchondromas, so any concern for a chondrosarcoma should be evaluated further. The chance of metastasis with chondrosarcomas involving the axial skeleton is much greater when compared to the appendages [4]. In a study done by Bovée et al., twenty-eight phalangeal chondrosarcomas were followed postoperatively from 8 to 432 months for recurrence and metastatic spread. Of the fifteen tumors treated with local curettage, ten recurred. Zero of the thirteen chondrosarcomas treated with radical surgery recurred. In this study, none of the phalangeal chondrosarcomas metastasized; thus, tumors involving the small bones of the hands and feet are rarely life threatening [8].

Treatment options for low-grade chondrosarcomas have been a recent topic for debate in the literature. The two treatment options that have been discussed are wide resection and intralesional curettage. Some authors/clinicians argue that wide excision is the only reasonable definitive treatment because of the metastatic potential of these tumors. Others comment that because of the benign likelihood of these lesions, intralesional curettage is the most appropriate option related to the reduced degree of morbidity and loss of function. In a systematic review done by Hickey et al., the authors were able to compare 190 patients from five separate studies, in which 112 patients underwent wide excision and 78 were treated with intralesional curettage. In this study, the risk of recurrence between the two groups was not significant [9].

In a recent study done by González del Pino et al., sixteen patients were evaluated for functional differences after either wide excision or intralesional curettage. Their data showed that of the nine patients (6 with primary lesions and 3 with recurrent disease) treated with curettage and bone grafting, two had recurrence. Of the eight patients treated with wide excision, one patient had a recurrence (patient was being treated for recurrent disease). In terms of functional status, there was no significant difference between groups when comparing grip strength, pinch strength, DASH (Disabilities of the Arm, Shoulder, and Hand) scores, and cosmesis. Limitations of the aforementioned study included a small sample size and lack of preoperative functional data. This indicates that there is a need for future studies on the functional outcomes for these treatments.

In our specific case, the patient had started to lose function with the adjacent digits and was no longer able to work. In addition to his functional loss, the patient's lesion was intra-articular, which made intralesional curettage a poor option. It was decided that ray resection would provide the patient with the best functional status postoperatively, while also reducing the chance of recurrent disease. As concluded by González del Pino et al., there are instances where intralesional curettage may be appropriate, but they believe that wide excision with disarticulation of the distal phalanges or digit ablation still plays a role in local control of the tumor while preserving function. In patients with obvious involvement of tendons, neurovascular compromise, or substantial deformity of neighboring joints, they call for the employment of amputation to preserve function [10]. We hope that this case was able to show some of the details involved with treatment of these tumors of the hand.
